# Unraveling the Coulombic Efficiency Trough of Silicon Anodes in Li‐Ion Batteries

**DOI:** 10.1002/smsc.202500131

**Published:** 2025-03-30

**Authors:** Asif Latief Bhat, Yu‐Sheng Su

**Affiliations:** ^1^ International College of Semiconductor Technology National Yang Ming Chiao Tung University 1001 Daxue Road Hsinchu 300093 Taiwan; ^2^ Industry Academia Innovation School National Yang Ming Chiao Tung University 1001 Daxue Road Hsinchu 300093 Taiwan

**Keywords:** fluoroethylene carbonate, irreversible loss, sponge structure, tetrahydrofuran, voltage windows, volume changes

## Abstract

The occurrence of Coulombic efficiency (CE) troughs in silicon (Si) anodes for lithium‐ion batteries (LIBs) presents a critical yet overlooked concern that can lead to battery failure in full cells. Herein, a comprehensive investigation into this previously unreported phenomenon is conducted. Factors influencing CE trough occurrence and severity, including electrode thickness, Si particle size, cycling rate, electrolyte composition, and voltage window, are systematically examined. Experimental results demonstrate that thinner electrodes and slower cycling rates accelerate CE trough onset, whereas employing a tetrahydrofuran (THF)‐based electrolyte or a narrower voltage window (0.01–0.5 V) results in stable electrochemical performance without CE troughs, concurrently with the presence of Li_*x*_Si. Structural analysis via high‐angle annular dark‐field scanning transmission electron microscopy and scanning electron microscopy reveals a close association between CE trough severity, electrode volume expansion, and delamination, influenced by the formation of a sponge‐like structure and solid electrolyte interface (SEI) stability. These findings yield valuable insights into CE trough mechanisms and provide guidance for mitigating their occurrence through electrode design, electrolyte selection, and operational parameters, thereby advancing high‐performance LIB development. Future research directions involve exploring the role of SEI components and alternative electrolyte additives to enhance SEI stability and mitigate CE troughs.

## Graphical abstract

The study systematically investigates the previously unreported coulombic efficiency (CE) trough phenomenon in silicon anodes for lithium‐ion batteries and identifies critical factors influencing CE trough occurrence, including electrode thickness, cycling rate, electrolyte composition, and voltage window. Structural analyses reveal that a sponge‐like structure emerges during CE trough, closely linked to electrode expansion, solid electrolyte interface instability, and reduced electrochemical performance.

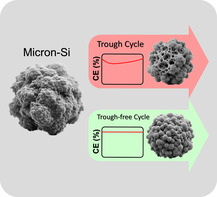

## 1 Introduction

The escalating demand for high‐energy‐density lithium‐ion batteries (LIBs) in smart grids and electric vehicles necessitates advancements in energy storage technology.^[^ ^1^, ^2^ ^]^ While graphite serves as a commercial anode material due to its high conductivity and reversibility, its limited capacity falls short of advanced LIB requirements.^[^ ^3^, ^4^ ^]^ Silicon (Si) emerges as a promising, earth‐abundant alternative, offering a tenfold higher specific capacity owing to its unique bonding with Li^+^ ions, enabling a theoretical capacity of 4200 mAh g^−1^.^[^ ^5^ ^]^ Despite these benefits, practical Si anode utilization faces challenges such as severe capacity fading and poor cycle life, attributed to volumetric changes (>300%) during lithiation and delithiation, resulting in particle pulverization, loss of electrical contact, and unstable solid electrolyte interface (SEI) formation.^[^ ^5^, ^6^ ^]^ The continuous degradation and renewal of the SEI film exacerbate these issues, hindering Li‐ion diffusion and increasing electrolyte consumption.^[^ ^5^, ^6^ ^]^ Various approaches, including nanostructuring Si, synthesizing composites, applying prelithiation, and employing new electrolyte formulations and binders, have been explored to mitigate these challenges, yielding excellent capacity retention over extended cycles.^[^ ^7^, ^8^, ^9^, ^10^, ^11^, ^12^, ^13^, ^14^, ^15^, ^16^, ^17^, ^18^, ^19^ ^]^

**Figure 1:**
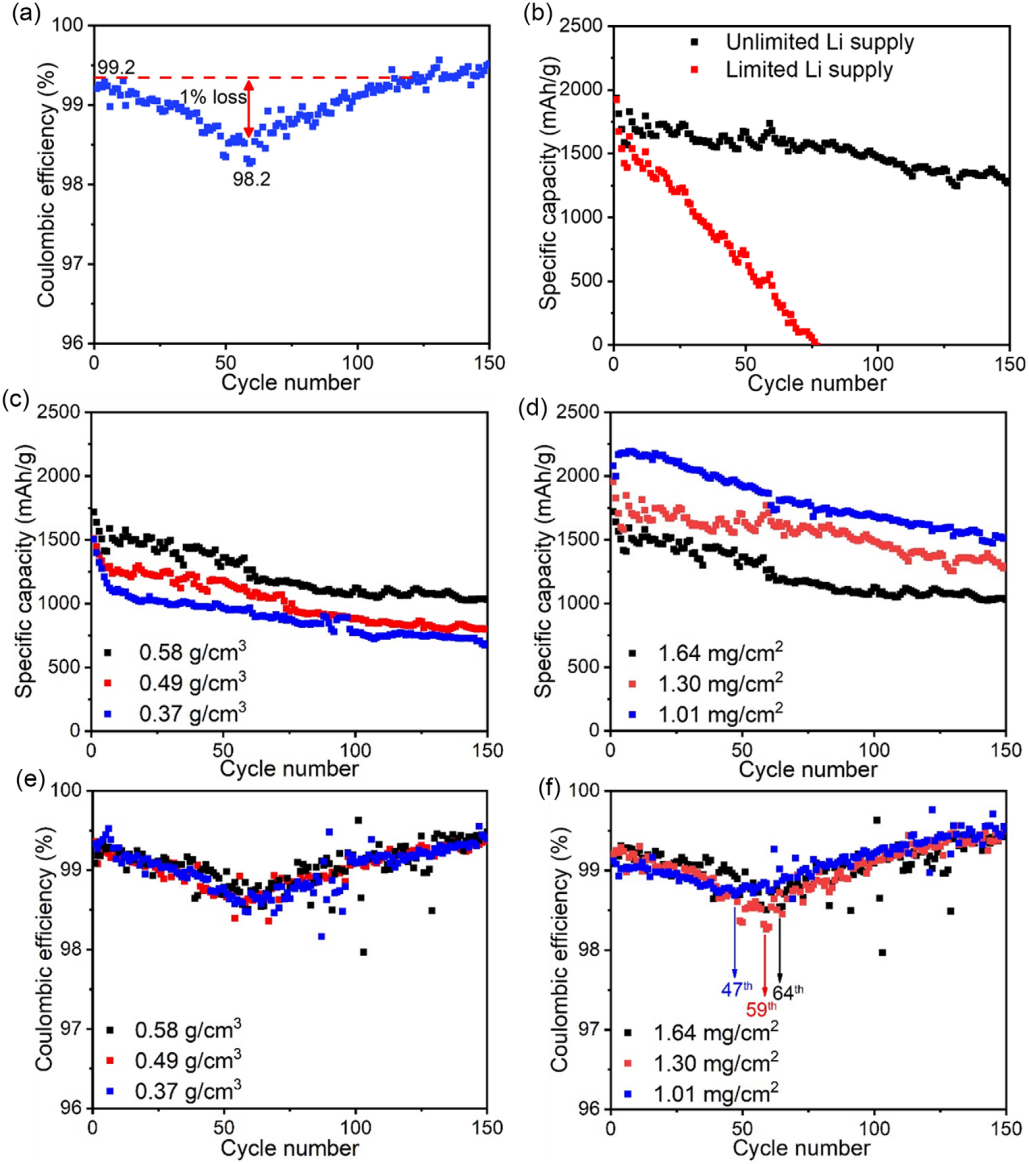
a) Typical CE trough of a Si anode during cycling. b) Cycling performance comparison of Si anodes with CE troughs in cells with unlimited or limited Li supply. Specific capacity variations of Si anodes with c) different packing densities and d) different areal densities. CE variations of Si anodes with e) different packing densities and f) different areal densities.

While extensive research has focused on enhancing the initial Coulombic efficiency (CE) of Si anodes through advanced prelithiation methods, surface modifications, and electrolyte additives,^[^ ^19^, ^20^, ^21^, ^22^, ^23^, ^24^, ^25^ ^]^ the long‐term evolution of CE during cycling remains relatively underexplored. In particular, the occurrence of CE troughs in later cycles has received little attention, despite their significant impact on full‐cell performance and cycle life. Bridging this gap, our study systematically investigates the underlying mechanisms driving CE trough formation and explores effective mitigation strategies. To the best of our knowledge, the CE trough phenomenon in Si anodes has not been previously reported in a systematic manner. As illustrated in **Figure** **1**a, a distinct decline in CE occurs in the early cycles, reaching its lowest point—a phenomenon we define as the CE trough. This trough poses a significant risk to battery performance, as it entails a loss of Li^+^ ions, with efficiency dropping from an initial 99.2% to 98.2%. This notable loss could potentially render the battery inoperative. Testing in a half‐cell setup with a nearly unlimited Li supply fails to reflect the capacity loss experienced during actual operation when the Li reservoir is limited (Figure 1b). This discrepancy highlights the importance of evaluating battery performance under realistic conditions to accurately assess capacity degradation. Similar troughs in Si anodes can be observed in several studies: Choi et al. reduced nanostructured SiO_2_ to nanoporous Si, revealing a CE trough;^[^ ^26^ ^]^ Cui et al. converted rice husks into Si nanoparticles and developed a pomegranate‐like Si anode, both exhibiting CE troughs;^[^ ^27^, ^28^ ^]^ Gasteiger et al. investigated Si–graphite anodes, noting larger troughs with higher Si content and smaller troughs with higher graphite content.^[^ ^29^ ^]^ These results underline the need to address and understand CE troughs in Si anodes for enhanced battery performance.

This research article delves into the underlying causes of the CE trough and proposes potential remedies. We investigate the effects of different areal and packing densities, C‐rates, Li metal/electrolyte content and freshness, as well as aging on this phenomenon. Additionally, internal and external pressures were applied to coin cells to assess their impact on the CE trough in Si anodes. Our comprehensive study employs various characterizations, including high‐angle annular dark‐field scanning transmission electron microscopy (HAADF‐STEM) with energy‐dispersive X‐ray spectroscopy (EDS), cross‐sectional scanning electron microscopy (SEM), dilatometer analysis for thickness changes, electrochemical impedance spectroscopy (EIS) analysis, and depth‐profiling X‐ray photoelectron spectroscopy (XPS) to elucidate the mechanisms driving this CE trough and explore potential solutions.

## 2 Results and Discussion

### 2.1 Factors Altering the Coulombic Efficiency Trough of Silicon Anodes

Figure 1c–f illustrates the impact of various packing and areal densities in Si anodes. Increased packing density enhances specific capacity by facilitating better contact among the active material, conductive agent, and current collector, thus improving electron transfer (Figure 1c). However, no variation in the CE trough is observed irrespective of packing density (Figure 1e). Conversely, areal density affects both specific capacity and the CE trough (Figure 1d,f). Higher areal density leads to decreased specific capacity due to longer electron/ion transfer paths in thicker electrodes. Notably, the CE trough manifests earlier and improves with lower areal density, as evidenced by the higher CE trough point observed in Figure 1f. Thus, while electrode capacity and packing density show no correlation with the CE trough, areal density appears to influence it, suggesting that thinner electrodes may marginally mitigate the CE trough and electrode failure.

**Figure 2:**
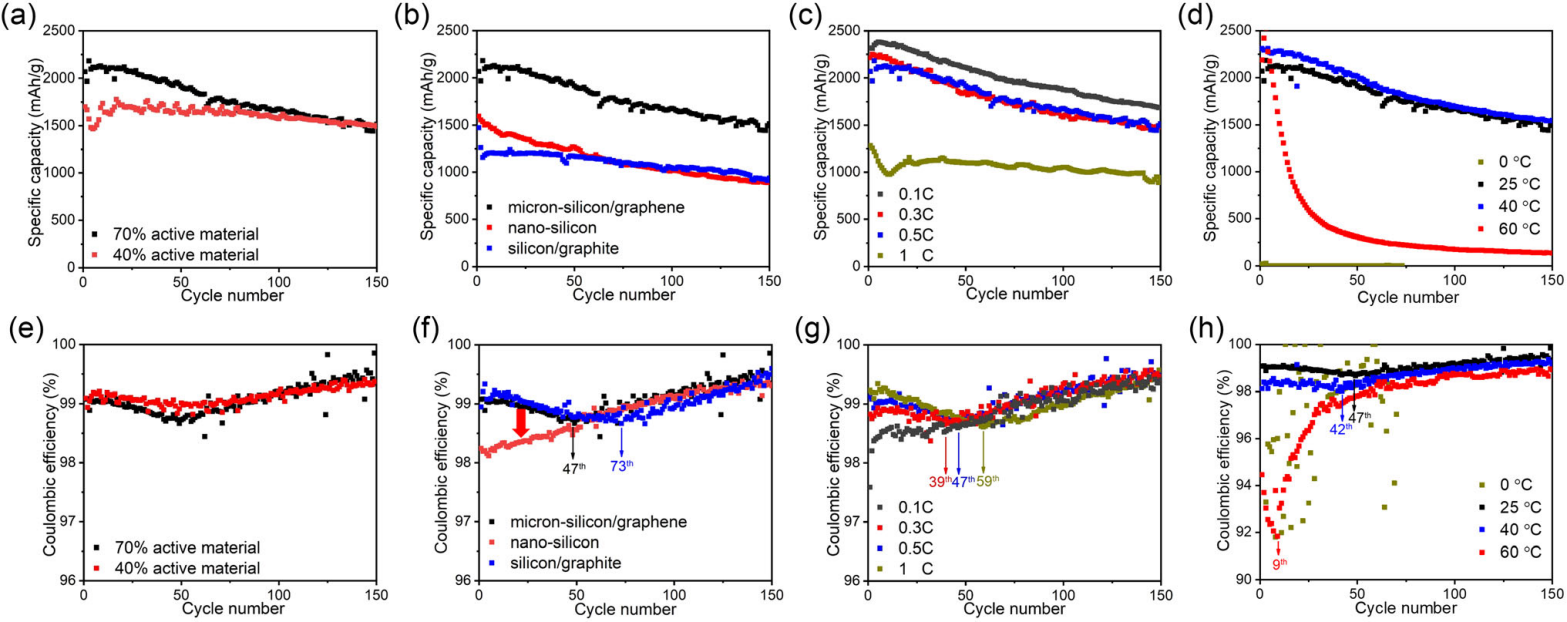
Specific capacity variations of Si anodes a) with 70% and 40% active material, b) using micron‐Si/graphene, nanosilicon, and Si/graphite composite materials, c) at different C‐rates, and d) at different temperatures. CE variations of Si anodes e) with 70% and 40% active material, f) using micron‐Si/graphene, nanosilicon, and Si/graphite composite materials, g) at different C‐rates, and h) at different temperatures.

Recognizing the variability of the CE trough under different conditions, we conducted a comprehensive analysis to elucidate the underlying mechanisms. **Figure** **2** illustrates the impact of various factors on the electrochemical performance of Si anodes. When utilizing 40% active material, as opposed to 70%, a more stable capacity retention can be observed, with the specific capacity reaching 1515 mAh g^−1^ after 150 cycles (Figure 2a). Moreover, the CE trough slightly improves with the 40% active material (Figure 2e), possibly attributed to the higher binder content promoting stronger adhesion between the active material and current collector, thereby minimizing volume expansion and enhancing cycle life. Comparisons between micron‐Si/graphene and nano‐Si electrodes (Figure 2b,f) reveal that nano‐Si exhibits a more pronounced CE trough at the beginning of cycling, suggesting that smaller particle size accelerates its onset. To further explore material effects, we synthesized a Si/graphite composite, commonly used in high‐capacity anodes. This composite exhibits a deeper CE trough than micron‐Si/graphene, reaching its minimum around the 73rd cycle, suggesting more severe active lithium loss. This behavior is likely attributed to the rigid graphite structure amplifying internal stress upon lithiation, unlike the more flexible graphene network, which better accommodates volume changes.

To investigate the correlation between the particle size and interfacial diffusion kinetics, we analyzed Li‐ion transport properties using EIS (Figure S1, Supporting Information). The Li‐ion diffusion coefficients (*D*_Li+_) were derived from the Warburg impedance in the low‐frequency region.^[^ ^30^ ^]^ Micron‐Si/graphene exhibits a higher *D*_Li+_ at the 5th cycle (3.34 × 10^−11^ cm^2^ s^−1^), which declines significantly to 3.14 × 10^−12^ cm^2^ s^−1^ by the 47th cycle, coinciding with the CE trough. This reduction is likely due to progressive structural deformation and SEI buildup. In contrast, nano‐Si displays consistently lower diffusion values from the outset, decreasing from 8.75 × 10^−13^ cm^2^ s^−1^ (5th cycle) to 3.71 × 10^−13^ cm^2^ s^−1^ (47th cycle). This early reduction is attributed to its higher grain boundary density, which promotes extensive SEI formation, increases interfacial resistance, and further hinders Li‐ion transport.

Beyond material properties, electrochemical cycling conditions also significantly impact CE trough behavior. Variations in C‐rate influence both capacity and trough onset (Figure 2c,g), with slower rates (e.g., 0.3 C) allowing more complete lithiation but leading to an earlier trough appearance (39th cycle) compared to faster rates (e.g., 1C, 59th cycle). This suggests that both Li^+^ diffusion behavior and reaction kinetics are key determinants of the CE trough. Furthermore, temperature plays a critical role in shaping CE trends (Figure 2d,h). Elevated temperatures (40 and 60 °C) accelerate the CE trough's onset due to increased electrolyte decomposition and SEI instability,^[^ ^31^, ^32^ ^]^ with the trough appearing as early as the 9th cycle at 60 °C. In contrast, at 0 °C, Li‐ion transport is significantly hindered, and SEI resistivity increases, leading to poor overall performance.^[^ ^31^ ^]^ Although moderate temperatures (e.g., 40 °C) initially enhance capacity by improving Li^+^ transport, they also accelerate active lithium consumption, ultimately reducing long‐term cycling CE.

Next, we explored several factors including Li supply by doubling Li foils, internal cell pressure by doubling springs, and electrolyte quantity by adjusting the injection volume during cell assembly. However, as illustrated in Figure S2a,d, Supporting Information, doubling the Li supply does not alter the CE trough in Si anodes, with the specific capacity showing a consistent trend compared to single Li foil usage. Although the application of two springs increases mechanical stress on battery components, particularly the Si anode experiencing substantial volume changes during cycling, it does not affect the CE trough. Instead, it leads to reduced available capacity during cycling, likely due to constrained expansion of the Si electrode (Figure S2b,e, Supporting information). Further investigation involving varying electrolyte volumes (50, 100, 150 μL) demonstrates a correlation between increased electrolyte volume and higher capacity, yet it remains independent of the CE trough, as shown in Figure S2c,f, Supporting Information. These findings suggest that the primary cause of the CE trough lies within the Si material itself rather than being attributed to deficiencies in Li‐ion supply, external stress, or electrolyte quantity.

Figure S3a,b, Supporting Information, illustrates the outcomes of two approaches aimed at addressing the CE trough. Initially, after identifying the trough cycle (≈50 cycles, indicated by the blue dashed line), we replaced the cycled Li foil, refreshed the aged electrolyte, and reassembled the coin cell with fresh components. Despite these efforts, the CE trough persists, as evidenced in Figure S3a, Supporting Information. Subsequently, we applied additional external pressure using a crimping machine after reaching the trough cycle, reducing the thickness of the coin cell, which had expanded due to Si anode swelling, to its original precycling dimensions (Figure S3b, Supporting information). However, this intervention fails to eliminate the CE trough, indicating that external pressure alone is unable to resolve the issue. Additionally, a 12 day rest period was conducted for the cell with Si anodes to observe any spontaneous changes in the CE trough behavior, yet no discernible difference was observed (Figure S3c,d, Supporting Information). These results suggest that alternative strategies beyond Li foil replacement, external pressure application, or extended resting periods are required to mitigate the CE trough phenomenon. Furthermore, these results reaffirm that the CE trough is unrelated to the aging of Li foil or electrolyte, as well as internal cell pressure.

**Figure 3:**
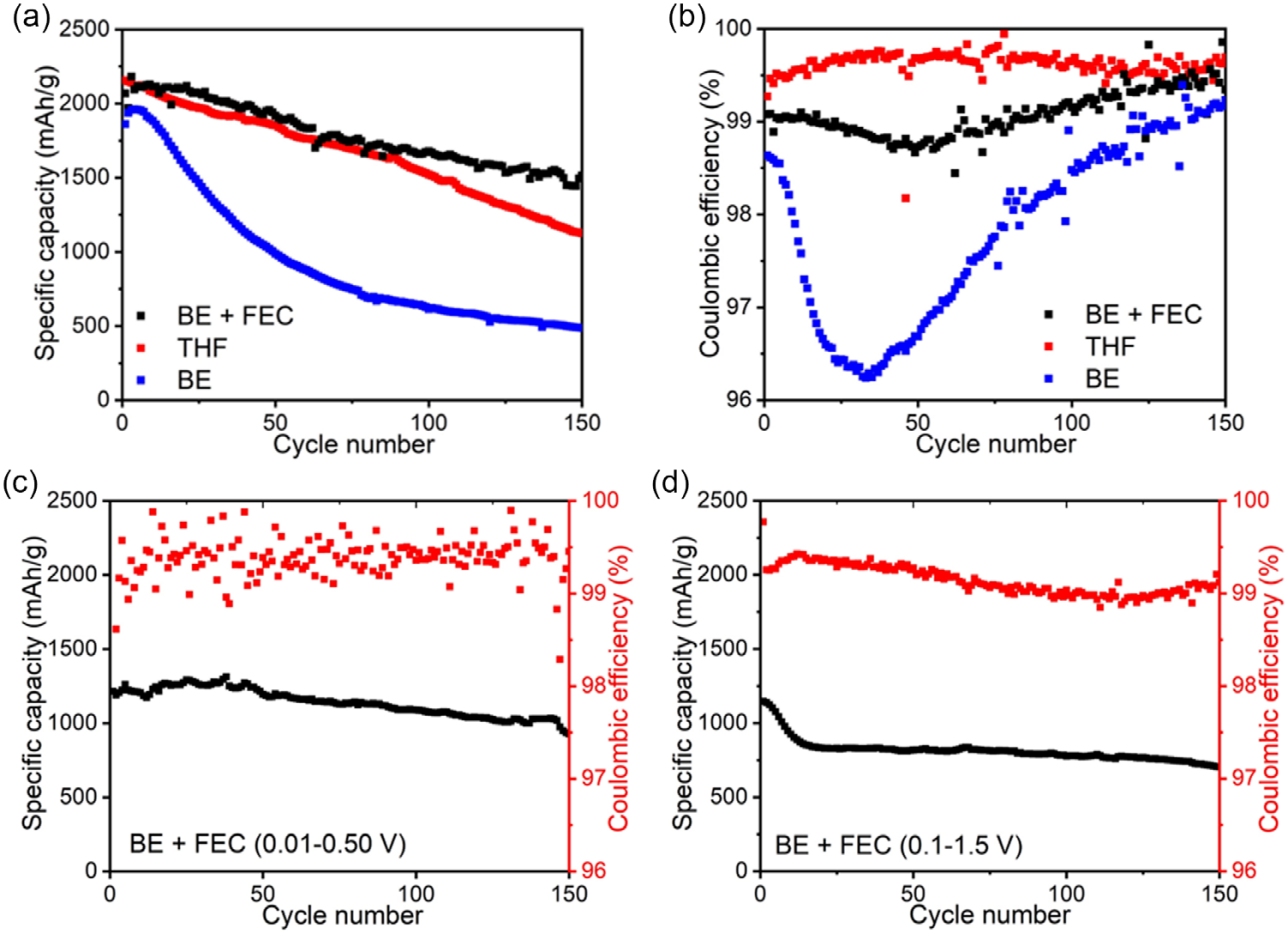
a) Specific capacity variations of Si anodes with different electrolytes. b) CE variations of Si anodes with different electrolytes. c) Specific capacity and CE of Si anodes cycled within 0.01–0.5 V. d) Specific capacity and CE of Si anodes cycled within 0.1–1.5 V.

Having explored how silicon particle size, cycling rate, and temperature can trigger or accelerate the CE trough, we next examined the role of electrolyte composition. Specifically, we evaluated how different electrolytes influence SEI stability and surface reactions, which ultimately impact electrode morphology and the severity of the CE trough. To assess the impact of electrolyte compositions on the CE trough phenomenon, we investigated three different electrolytes: BE (base carbonate electrolyte), BE + FEC (with fluoroethylene carbonate (FEC) additive), and THF‐based electrolyte).^[^ ^33^ ^]^ Notably, with BE electrolyte, a notable capacity drop can be observed after a few cycles (**Figure** **3**a). This decline can be attributed to the formation of a thick and unstable SEI layer on the Si anodes, a well‐documented phenomenon in literature.^[^ ^34^ ^]^ Figure 3b illustrates a more pronounced CE trough with BE electrolyte compared to the other two electrolytes, primarily due to extensive volume expansion and unstable SEI formation. In contrast, the utilization of BE + FEC electrolyte leads to an improved cycle life and a mitigated CE trough compared to BE electrolyte. Remarkably, when THF electrolyte was employed, no CE trough was observed. This could be attributed to the promotion of a thin and robust LiF‐rich SEI with high mechanical strength by THF electrolyte.^[^ ^35^ ^]^ Consequently, the properties of the SEI appear to be closely correlated with the behavior of CE trough.

Subsequently, we investigated how the state of charge and discharge (SoC and SoD) can influence the CE trough by modifying the voltage window to 0.01–0.5 V (high SoC and low SoD) and 0.1–1.5 V (low SoC and high SoD).^[^ ^36^ ^]^ Utilizing the cyclic voltammogram (CV) analysis (Figure S4, Supporting Information), we set the cutoff voltages at 0.5 and 0.1 V to partially delithiate and lithiate the Si anodes, respectively. With the 0.01–0.5 V window, complete lithiation and partial delithiation are achieved, ensuring a stable cycle life and the absence of the CE trough (Figure 3c). However, minor CE fluctuations were observed, likely due to residual side reactions and interfacial instabilities, which require further investigation. Conversely, incomplete lithiation followed by complete delithiation using the 0.1–1.5 V window results in an initial degradation in specific capacity, followed by stable capacity retention and the occurrence of the CE trough at the 120th cycle (Figure 3d). These findings demonstrate that maintaining the Si anodes fully lithiated and partially delithiated can be a viable means to improve electrochemical stability, not only in terms of capacity retention but also by reducing irreversible loss, as evidenced by the absence of the CE trough. Overall, both electrolyte composition and voltage window strongly influence SEI formation and stability, which, in turn, governs electrode surface evolution during cycling. To highlight the unique contributions of our study, we have compiled a comparative analysis in Table S1, Supporting Information, which contrasts key experimental conditions, material compositions, electrochemical results, and key findings across prior studies and our current work. By directly comparing these parameters, we illustrate how our methodology provides new insights into CE trough mitigation.

### 2.2 Structural Change of Silicon Anodes after Trough Cycle

**Figure 4:**
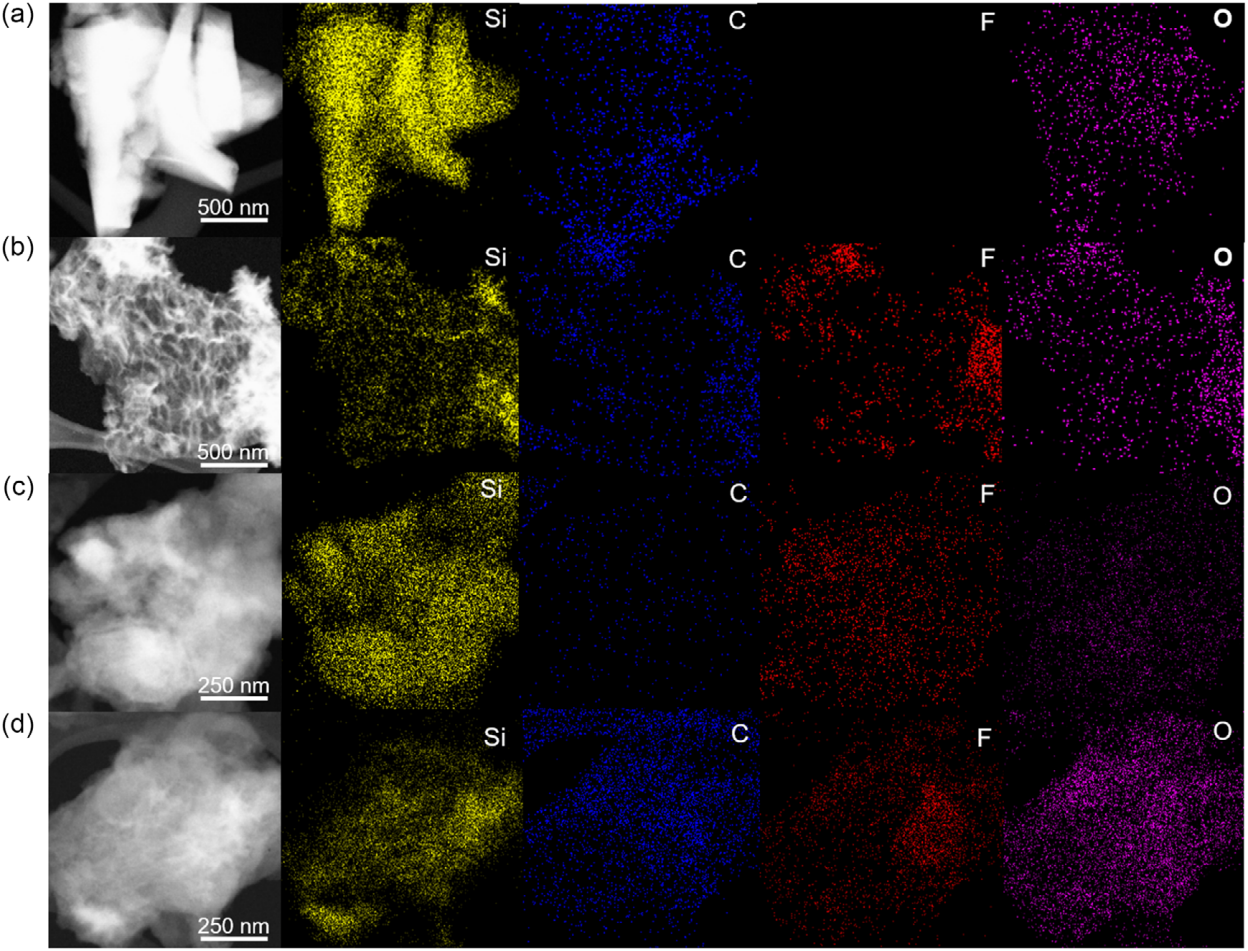
HAADF‐STEM with EDS mapping images of Si anodes a) before cycling, b) after the CE trough cycle with BE + FEC electrolyte and 0.01–1.5 V, c) after the CE trough cycle with THF electrolyte and 0.01–1.5 V, and d) after the CE trough cycle with BE + FEC electrolyte and 0.01–0.5 V.

To understand these observations more deeply, we next explored the corresponding structural changes and morphology of Si anodes, linking the observed electrochemical behavior—particularly the presence or absence of the CE trough—to underlying physical transformations. In **Figure** **4**, HAADF‐STEM and EDS mapping images of Si electrodes before and after cycling are displayed. Initially, a dense crystalline structure is observable on the Si particles prior to cycling, as shown in Figure 4a. Upon cycling with BE + FEC electrolyte under the full voltage window (0.01–1.5 V) until reaching the CE trough (47th cycle), a significant transformation is observed: the Si structure adopts a highly porous and sponge‐like morphology, accompanied by a notable decrease in density attributed to volume expansion, as illustrated in Figure 4b. Analysis of the Si signals (yellow) indicates a pronounced reduction in Si density within the expanded particles, indicative of highly porous and amorphous structure formation. Conversely, when employing THF electrolyte, a denser postcycling structure is maintained compared to BE + FEC electrolyte, suggesting lesser volume expansion and more stable SEI formation, as observed in Figure 4c. Its carbon and oxygen spectra exhibit minimal abundance, implying unfavorable Li_2_CO_3_ and Li_2_O formation, which are common SEI components in carbonate‐based electrolytes. Furthermore, the relatively dense Si structure is also retained when cycled with BE + FEC electrolyte and a narrowed 0.01–0.5 V voltage window, as shown in Figure 4d, with fluorine, oxygen, and carbon spectra exhibiting comparable abundance levels. Although a decrease in density is observed, the absence of the sponge‐like morphology suggests that restricting the upper cutoff voltage plays a crucial role in preventing extreme structural degradation. These observations indicate that the sponge‐like transformation in Si anodes is closely tied to the voltage window and electrolyte selection. Specifically, the absence of this morphology in Si anodes cycled with THF electrolyte or an early 0.5 V cutoff potential likely explains why these conditions prevent the occurrence of CE troughs. Additional HAADF‐STEM images from various locations are provided in Figure S5, Supporting Information.

**Figure 5:**
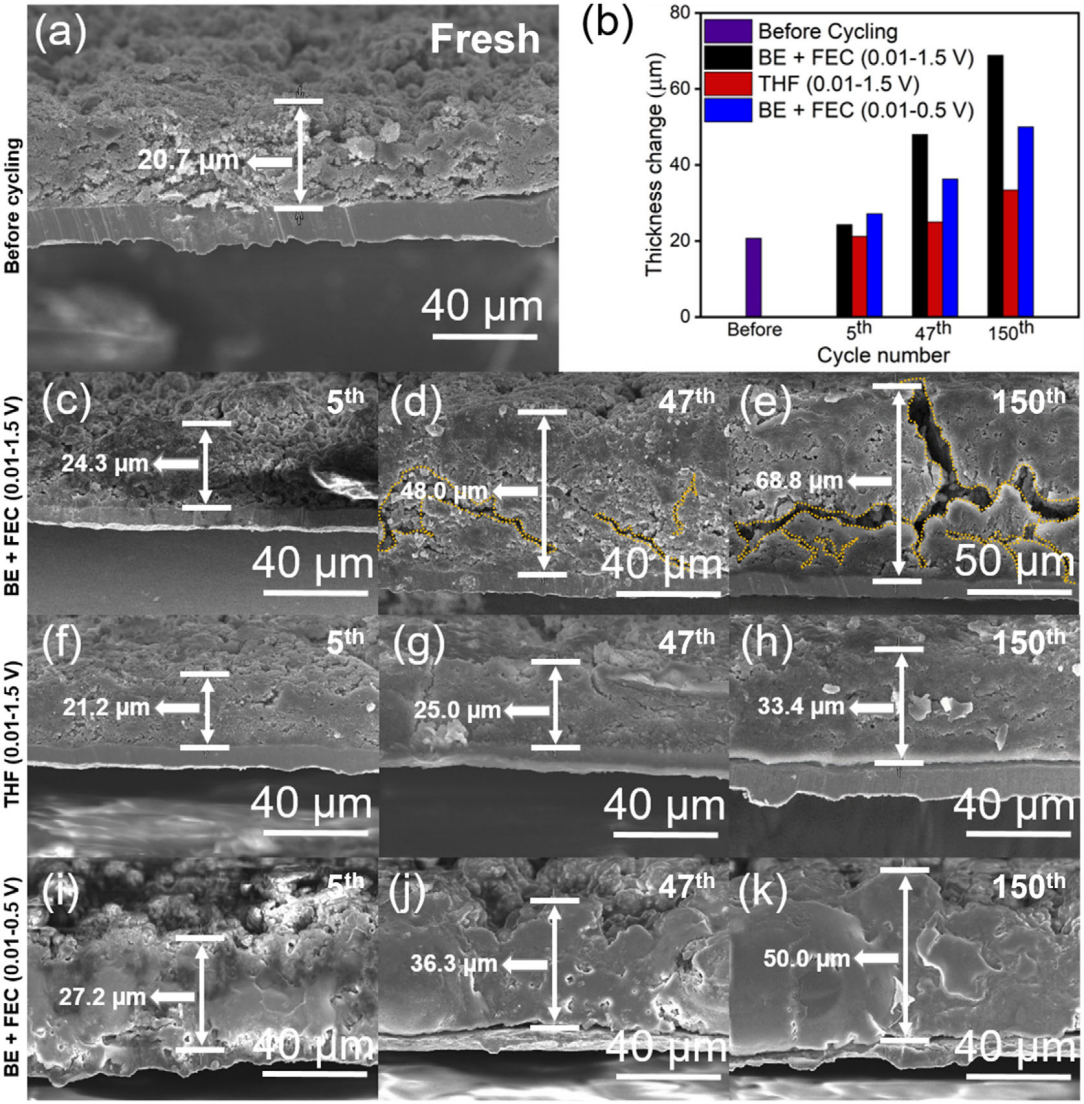
a) Cross‐sectional SEM image of Si anode before cycling. b) Thickness change with different electrolytes and voltage windows at different cycle numbers. Cross‐sectional SEM images of Si anode cycled with c–e) BE + FEC electrolyte and 0.01–1.5 V, f–h) THF electrolyte and 0.01–1.5 V, and i–k) BE + FEC electrolyte and 0.01–0.5 V at the 5th, 47th (the CE trough cycle), and 150th cycles, respectively.

**Figure** **5** illustrates SEM cross sections of Si anodes both before and after cycling, accompanied by a bar plot (Figure 5b) showing the considerable volume expansion of Si anodes cycled with BE + FEC electrolytes under a full voltage window (Figure 5c–e). In contrast, the utilization of either THF electrolyte or BE + FEC electrolyte with a 0.01–0.5 V window demonstrates effective mitigation of this expansion (Figure 5f–k), thereby preserving SEI integrity. Notably, severe cracking of Si anodes cycled with BE + FEC electrolyte at 0.01–1.5 V initiates at the 47th cycle (Figure 5d), with pronounced cracks evident after the 150th cycle (Figure 5e). Conversely, Si anodes cycled with THF electrolyte or BE + FEC electrolyte at 0.01–0.5 V exhibit a fissure‐free morphology (Figure 5f–k), with the smallest swelling observed in Si anodes using THF electrolyte, thickening from 20.7 to 33.4 μm. Supporting these observations, Figure S6, Supporting Information, provides additional insights with dilatometer data, comparing Si anode volume expansion cycled with 0.01–1.5 and 0.01–0.5 voltage windows. In Figure S6a, Supporting Information, substantial volume expansion occurs up to the CE trough cycle, followed by a post‐CE trough cycle decrease, resulting in a total volume expansion of nearly 240% after 150 cycles. The slopes before and after the CE trough are 2.09% and 1.24% per cycle, respectively, indicating a decline in volume expansion rates after the CE trough. Figure S6b, Supporting Information, presents swelling data with a narrowed 0.01–0.5 V voltage window, revealing 137% volume expansion after 150 cycles with a markedly smaller slope of 0.89% per cycle. These findings highlight the absence of a CE trough, strongly correlating with controllable volume change behavior of Si anodes using THF electrolyte or a 0.01–0.5 V cycling window.

### 2.3 Electrochemical and Chemical Analysis of Silicon Anodes after Trough Cycle

**Figure 6:**
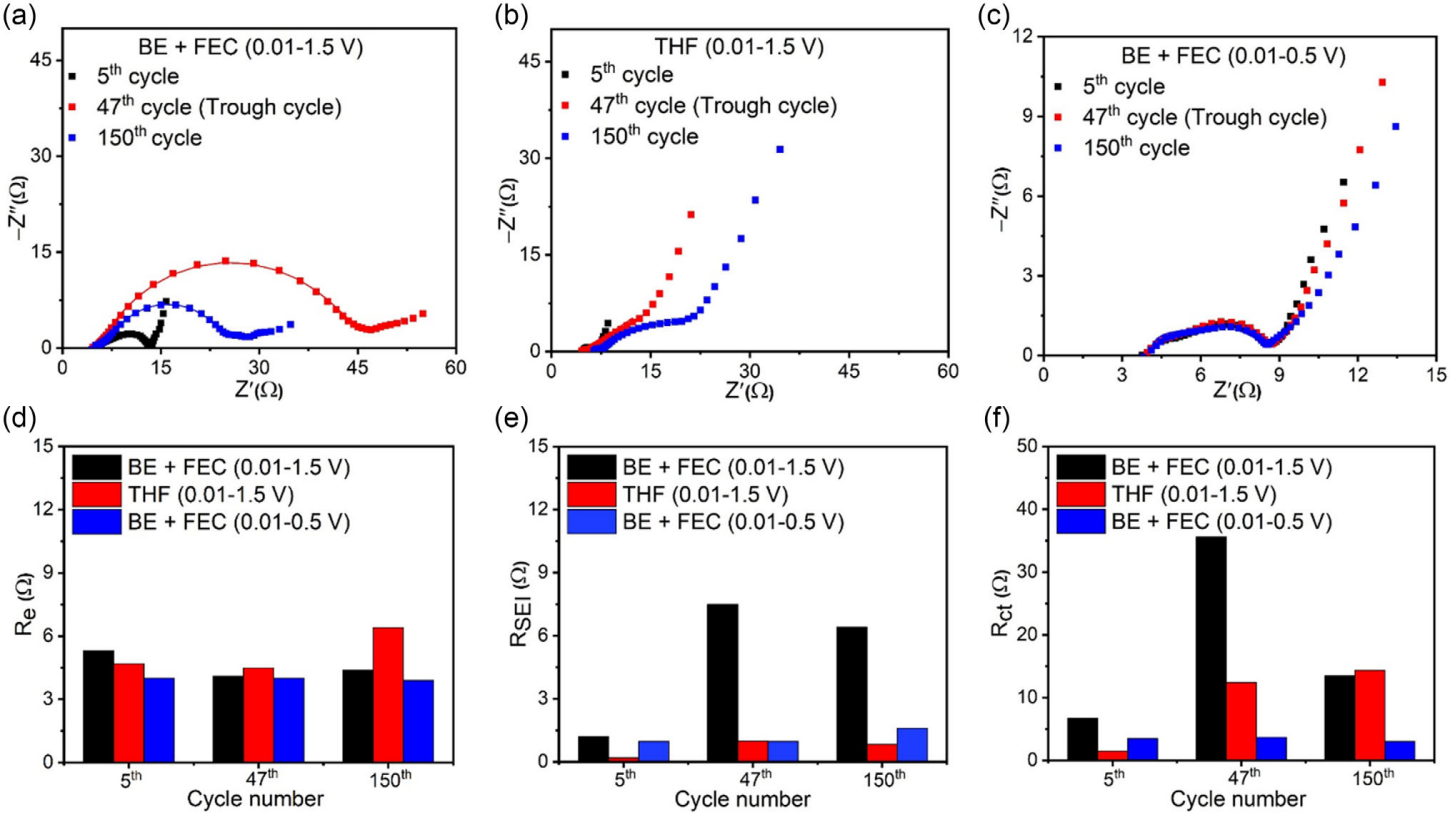
EIS spectra of Si anodes cycled with a) BE + FEC electrolyte and 0.01–1.5 V, b) THF electrolyte and 0.01–1.5 V, and c) BE + FEC electrolyte and 0.01–0.5 V at the 5th, 47th (the CE trough cycle), and 150th cycles. Comparison of d) *R*_e_, e) *R*_SEI_, and f) *R*_ct_ resistances with different electrolytes and voltage windows.

**Figure** **6** illustrates the outcomes of EIS tests performed on Si anodes cycled with different electrolytes and voltage windows at the 5th, 47th, and 150th cycles. The equivalent circuit parameters outlined in Figure S7, Supporting Information, encompass *R*_e_ for electrolyte resistance, *R*_SEI_ for SEI resistance, and *R*_ct_ for charge transfer resistance.^[^ ^37^, ^38^ ^]^ Notably, SEI resistance (Figure 6e) exhibits substantially higher values at the 47th and 150th cycles with BE + FEC electrolyte cycled under the full voltage window, indicating thick and unstable SEI formation. In contrast, THF electrolyte or a narrowed 0.01–0.5 voltage window leads to notably smaller and stable SEI resistance, suggesting improved interfacial stability. Charge transfer resistance trends (Figure 6f) further support these observations. BE + FEC electrolyte with a 0.01–1.5 V window leads to a sharp *R*_ct_ increase at the 47th cycle, consistent with the sponge‐like morphology observed in Figure 4b, which disrupts charge transport. Conversely, THF electrolyte and a 0.01–0.5 V voltage window promote the retention of partially delithiated Li_*x*_Si, which enhances electronic/ionic conductivity and suppresses *R*_ct_ growth over cycling.^[^ ^36^ ^]^ Notably, *R*_ct_ is lowest at the 5th cycle, when the silicon anode structure remains relatively intact, maintaining efficient charge transport pathways. However, lithiation‐induced expansion leads to structural degradation, SEI thickening, and increased *R*_ct_ in later cycles. The THF electrolyte and a restricted voltage window (0.01–0.5 V) effectively suppress these increases, stabilizing the electrode–electrolyte interface and preserving conductive Li_*x*_Si phases, thereby reducing impedance over extended cycling.

**Figure 7:**
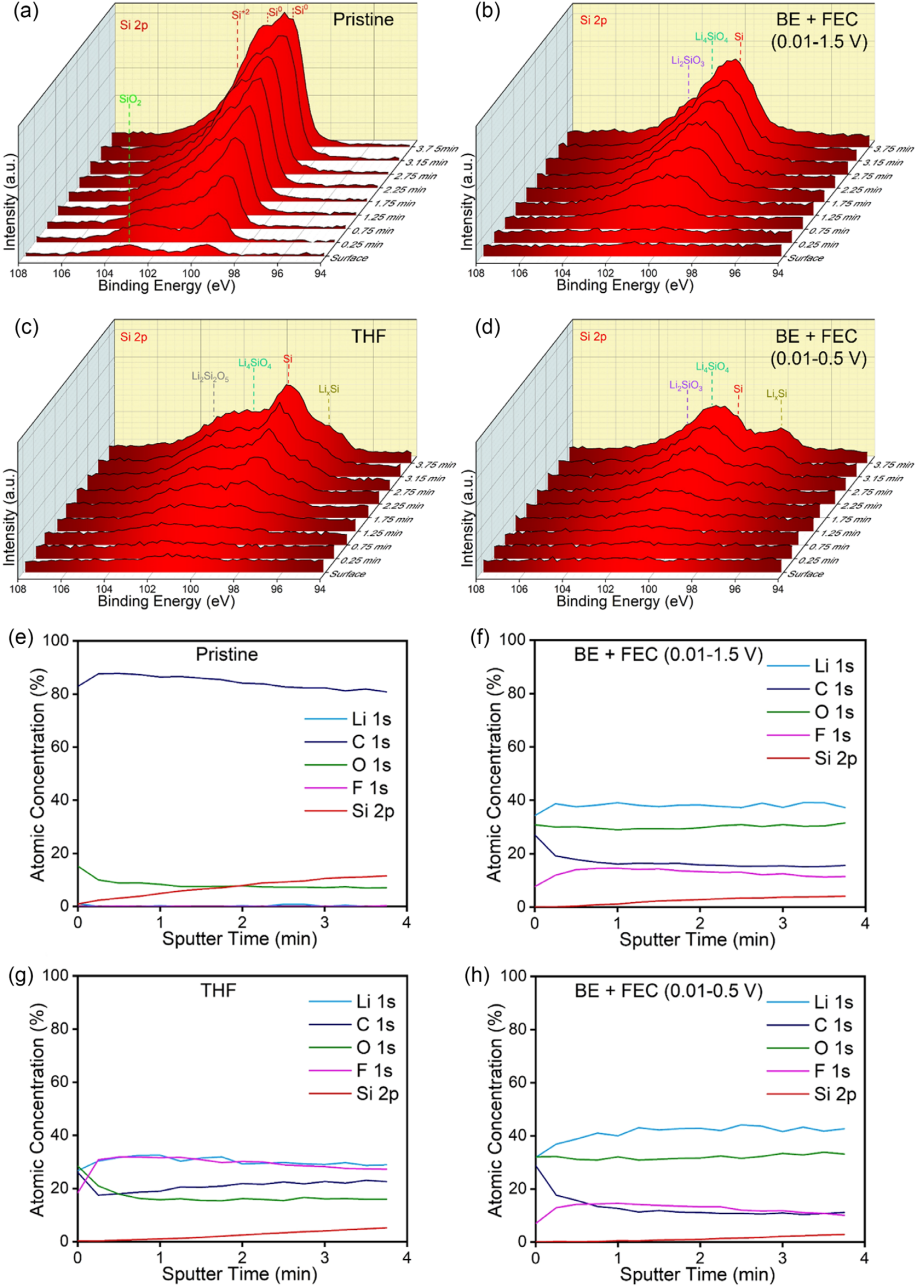
XPS Si 2*p* depth profiles of Si anodes a) before cycling, at the CE trough cycle with b) BE + FEC electrolyte and 0.01–1.5 V, c) THF electrolyte and 0.01–1.5 V, and d) BE + FEC electrolyte and 0.01–0.5 V. Atomic distribution of Si anodes e) before cycling, at the CE trough cycle with f) BE + FEC electrolyte and 0.01–1.5 V, g) THF electrolyte and 0.01–1.5 V, and h) BE + FEC electrolyte and 0.01–0.5 V versus sputter time.

To investigate how the interfacial compositions of Si anodes with different electrolytes and voltage windows change after cycling, **Figure** **7** and S8–11, Supporting Information, present depth profiling analysis conducted via XPS for Si 2*p*, O 1*s*, C 1*s*, F 1*s*, and Li 1*s* spectra, respectively, on Si anodes before and after cycling. The fresh Si spectra in Figure 7a exhibit peaks corresponding to Si and surface native oxides, with the intensity of Si^0^ increasing with the depth of pristine Si.^[^ ^39^, ^40^ ^]^ These Si peaks persist after cycling with BE + FEC and THF electrolytes, displayed in Figure 7b–d. After the CE trough cycle, lithium silicate peaks form in Si 2*p* and O 1*s* with both BE + FEC and THF electrolytes as shown in Figure 7b–d and S8b–d, Supporting Information, with Li_4_SiO_4_ and Li_2_SiO_3_ peaks evident with BE + FEC electrolyte, and Li_4_SiO_4_ and Li_2_Si_2_O_5_ peaks observed with THF electrolyte.^[^ ^35^, ^41^, ^42^, ^43^, ^44^ ^]^ Li_2_CO_3_ and Li_2_O are the main SEI components in the carbonate‐based electrolyte (BE + FEC), and LiOH replaces Li_2_CO_3_ as the major SEI in the THF electrolyte,^[^ ^35^, ^45^ ^]^ along with less Li_2_O formation (Figure S8c, Supporting Information).^[^ ^35^, ^43^, ^44^ ^]^ Notably, additional Li_*x*_Si peaks appear for cells with THF electrolyte and 0.01–0.5 V,^[^ ^35^, ^42^ ^]^ which exist without the CE trough. The preservation of Li_*x*_Si, a better electrical and ionic conductor than pristine Si, can be crucial to maintaining high CE during cycling. Figure S9a–d, Supporting Information, displays XPS spectra of C 1*s*, revealing the presence of C—C and C—O peaks before and after cycling with both BE + FEC and THF electrolytes,^[^ ^42^, ^45^, ^46^ ^]^ indicating signals from graphene, carbon black, and binders. F 1*s* and Li 1*s* spectra in Figure S10, 11, Supporting information, demonstrate LiF formation in both types of electrolytes,^[^ ^43^ ^]^ along with residual LiPF_6_ salt precipitated on the surface of porous electrode material.^[^ ^35^, ^43^ ^]^ Figure 7e–h compares atomic concentration at various depths, showing slightly more Li preserved within the narrow voltage window (0.01–0.5 V; Figure 7f) compared to the wide voltage window (0.01–1.5 V; Figure 7h), probably due to the existing Li_*x*_Si compound in the former case. On the other hand, the cell with THF electrolyte exhibits significantly higher F signals (Figure 7g and S10, Supporting Information), implying the strong formation of LiF, a sturdy low‐impedance SEI compound that is advantageous for stable cycling.^[^ ^35^ ^]^ These XPS results confirm that the formation of a mechanically robust, LiF‐rich SEI prevents extensive electrode cracking and sponge‐like pore formation, hence averting the characteristic CE trough.^[^ ^35^ ^]^ In contrast, incomplete or unstable SEI coverage promotes aggressive silicon corrosion and volume expansion, culminating in the efficiency dip observed as the CE trough.

**Scheme 1:**
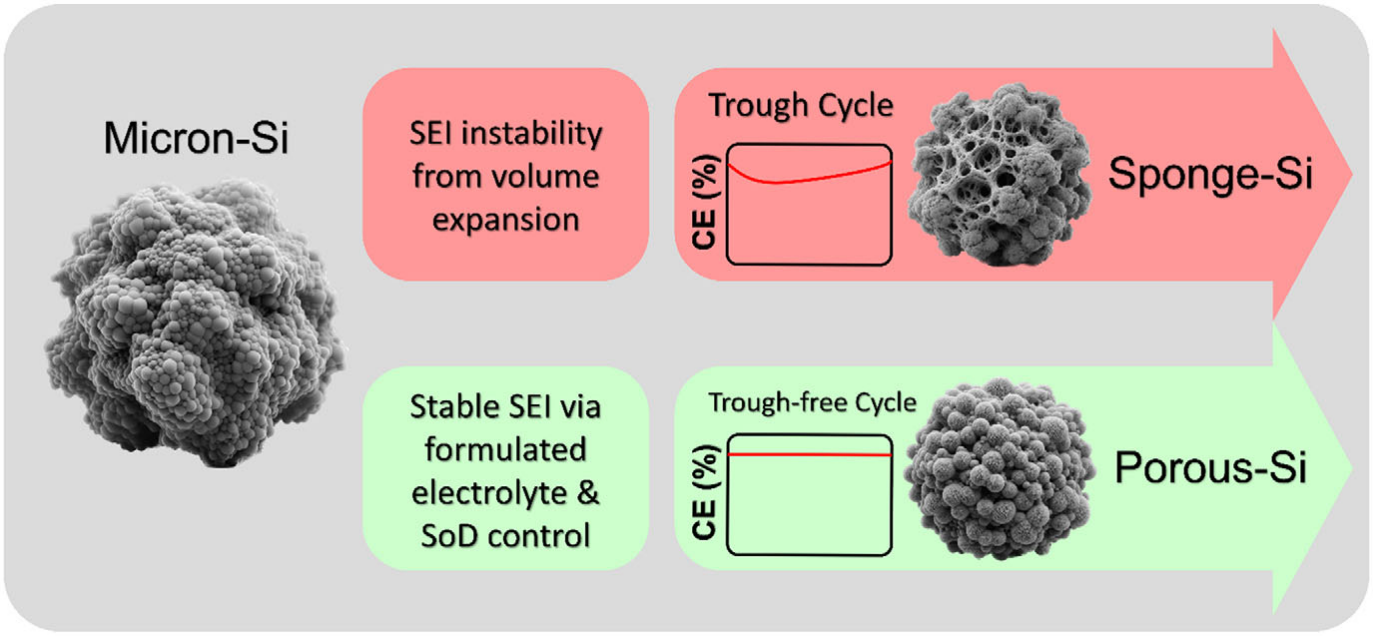
Structural changes in micron‐silicon anodes after the CE trough and trough‐free cycles.

Based on the analytical results in this study, we learned that when the SEI is not strong enough to withstand the repeated volume expansion of Si, the CE trough will occur, along with sponge‐like Si formation due to the corrosion led by repeated SEI formation, as shown in **Scheme** **1**. In contrast, if the SEI can maintain certain stability over cycling, either by the aid from the THF electrolyte that can promote LiF‐rich SEI formation or by the SoD control to avoid full delithiation, the electrode generates intraparticle pores with much better CE without trough. The former provides mechanically strong SEI protection and the latter offers smaller swelling, both contributing to the prevention of severe SEI damage. Additionally, the Li_*x*_Si compound always exists in the CE trough‐free cell, indicating the preservation of lithiated silicon could be beneficial for high‐efficiency anode reactions in LIBs.

## 3 Conclusions

In summary, this study highlights and investigates the previously unreported phenomenon of CE troughs in Si anodes of LIBs. Table S2, Supporting Information, summarizes both the factors that influence CE troughs and those that do not. Through a comprehensive experimental study, we identified key factors influencing the occurrence and severity of CE troughs, including electrode thickness, Si particle size, cycling rate, electrolyte composition, and voltage window. Thinner electrodes and slower cycling rates were found to accelerate the onset of CE troughs, while the utilization of THF electrolyte or a narrower voltage window (0.01–0.5 V) led to stable electrochemical performance without CE troughs. Structural analysis via HAADF‐STEM and cross‐sectional SEM revealed that the severity of CE troughs is closely linked to electrode volume expansion and delamination, influenced by the formation of a sponge‐like structure and SEI instability. Furthermore, the consistent presence of the Li_*x*_Si compound in CE trough‐free cells suggests that preserving lithiated silicon could offer benefits for high‐efficiency anode reactions in LIBs. These findings provide valuable insights into the mechanisms underlying CE troughs in Si anodes and offer guidance for mitigating their occurrence through electrode design, electrolyte selection, and operational parameters, thereby advancing the development of high‐performance LIBs. While this study successfully identifies the factors governing CE trough formation and suppression, further research is needed to address residual CE fluctuations and enhance long‐term cycling stability. Future work should focus on: 1) elucidating the role of SEI composition and stability, particularly investigating the formation of LiF‐rich SEI through XPS and in situ techniques; 2) exploring alternative electrolyte additives and interfacial engineering strategies to suppress residual CE fluctuations after CE trough elimination; and 3) leveraging advanced characterization techniques, such as in situ TEM and operando X‐ray spectroscopy, to gain real‐time insights into electrochemical and structural evolution during cycling. By addressing these challenges, this research lays the foundation for more efficient and durable Si‐based anodes, ultimately contributing to the development of next‐generation LIBs.

## 4 Experimental Section

4.1

4.1.1

##### Electrode Fabrication

The anode slurry comprised 70% micron‐Si/graphene (Angstron Energy Company) or nano‐Si (Silican) or Si/graphite composite active material, 10% conductive carbon black (Super P), 10% carboxymethyl cellulose (Dow), and 10% styrene–butadiene rubber (JSR). Unless otherwise specified, comparisons were conducted using the micron‐Si/graphene anode. Deionized water served as the medium during mixing, with stirring conducted for three hours. Subsequently, the slurry was tape‐cast onto a copper current collector and dried under vacuum at 80 °C for a minimum of 12 h. After drying, the sheet was rolled and precisely cut to generate electrodes with a diameter of 1.5 cm. The Si/graphite composite was synthesized by ball‐milling silicon and graphite (1:1 weight ratio) with zirconia balls for 48 h using IPA, followed by air‐drying (80 °C) and vacuum‐drying (100 °C).

##### Battery Assembly and Cell Testing

CR2032 coin cell assembly involved stacking the Si electrode, electrolyte, Celgard separator, and Li metal foil. Three types of electrolytes were used: BE (1.0 M LiPF_6_ in EC/EMC = 1:2 in vol), BE + FEC (BE electrolyte + 25 vol% FEC), and THF (2.0 M LiPF_6_ in THF/2mTHF = 1:1 in vol). Unless otherwise specified, comparisons were conducted using BE + FEC electrolyte. Assembly occurred in an Ar‐filled glove box with O_2_ and H_2_O levels <0.5 ppm. Electrochemical evaluations were conducted using a battery tester (Neware Technology) at 25 °C, with a cutoff voltage of 0.01 V versus Li^+^/Li for lithiation and 1.5 V for delithiation. The cell underwent cycling at 0.1 C‐rate for three formation cycles, followed by cycling at 0.5 C‐rate for subsequent cycles. CVs were recorded utilizing a potentiostat (BioLogic SP‐50e), with a voltage range of 0.01–1.5 V and a sweep rate of 0.1 mV s^−1^. EIS was performed on the same tester from 1 to 50 mHz with an AC voltage magnitude of 10 mV. The Warburg factor, which represents Li‐ion diffusion impedance at low frequencies, was determined by fitting the sloped region of the Nyquist plots and used to calculate the Li‐ion diffusion coefficient.^[^ ^30^ ^]^

##### Material Characterizations

HAADF‐STEM and EDS (JEOL ARM200F) were employed to analyze the morphology and relative composition of Si particles from uncycled electrodes and those cycled up to the CE trough cycle. For cycled sample preparation, Si electrodes were delithiated at 1.5 V vs. Li^+^/Li, harvested from the cells, washed with dimethyl carbonate, and dried. Cross‐sectional SEM (Hitachi SU‐8010) was utilized to examine the morphology and thickness of Si electrodes before and after cycling. Real‐time electrode thickness was measured using a cell‐type dilatometer (2E‐CELL‐ETC; Eager Corporation). Additionally, XPS (ULVAC‐PHI Quantera II) was employed for depth profile analysis of Si anodes, providing insights into composition variations at different locations.

## Conflict of Interest

The authors declare no conflict of interest.

## Author Contributions

**Asif Latief Bhat**: data curation (lead); formal analysis (lead); investigation (lead); methodology (supporting); validation (lead); visualization (supporting); writing—original draft (supporting); writing—review and editing (supporting). **Yu‐Sheng Su**: conceptualization (lead); funding acquisition (lead); investigation (equal); methodology (lead); project administration (lead); resources (lead); supervision (lead); validation (supporting); visualization (lead); writing—original draft (lead); writing—review and editing (lead).

## Supporting information

Supplementary Material

## Data Availability

The data that support the findings of this study are available from the corresponding author upon reasonable request.
