# Supplementary material for: Unraveling the Coulombic Efficiency Trough of Silicon Anodes in Li‐Ion Batteries
Source: Small Sci. 2025 Mar 30;5(7):2500131. doi: 10.1002/smsc.202500131 (PMC12257903; doi:10.1002/smsc.202500131)
Supplement: Supplementary file 1 — Supplementary Material [file SMSC-5-2500131-s001.pdf]

Unraveling the Coulombic Efficiency Trough of Silicon Anodes in Li-ion Batteries

Asif Latief Bhat[1] *and Yu-Sheng Su**[1,2]

[1]International College of Semiconductor Technology, National Yang Ming Chiao Tung University, 1001 Daxue Road, Hsinchu 300093, Taiwan

[2]Industry Academia Innovation School, National Yang Ming Chiao Tung University, 1001 Daxue Road, Hsinchu 300093, Taiwan

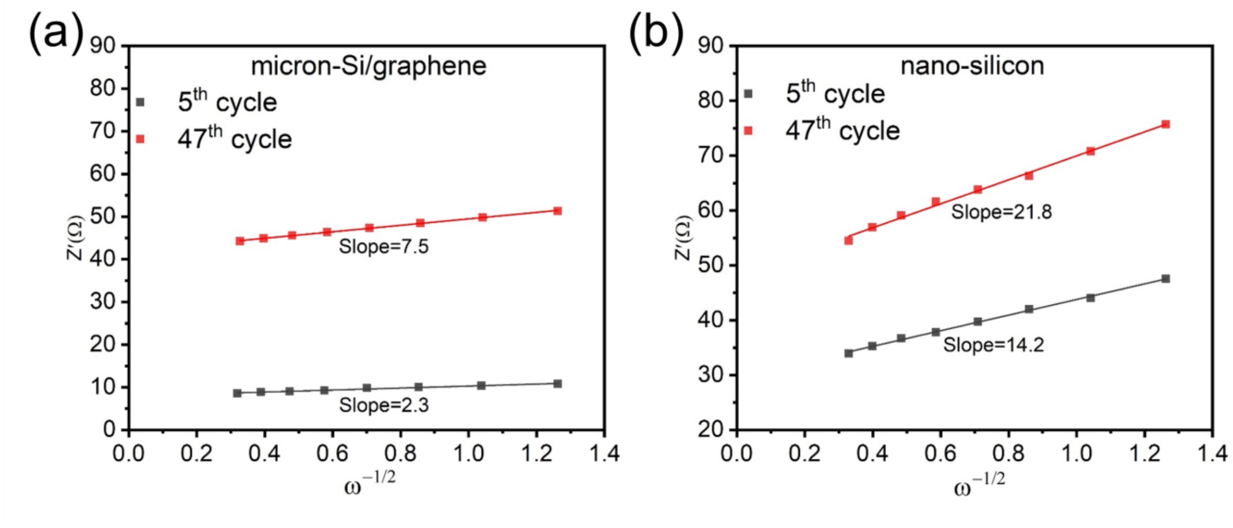

Figure S1. Warburg impedance-derived Li-ion diffusion analysis for Si anodes. Linear relationship between Warburg impedance in the low-frequency region and the inverse square root of angular frequency, where the slope represents the Warburg factor for (a) micron-Si/graphene and (b) nano-silicon at different cycle numbers.

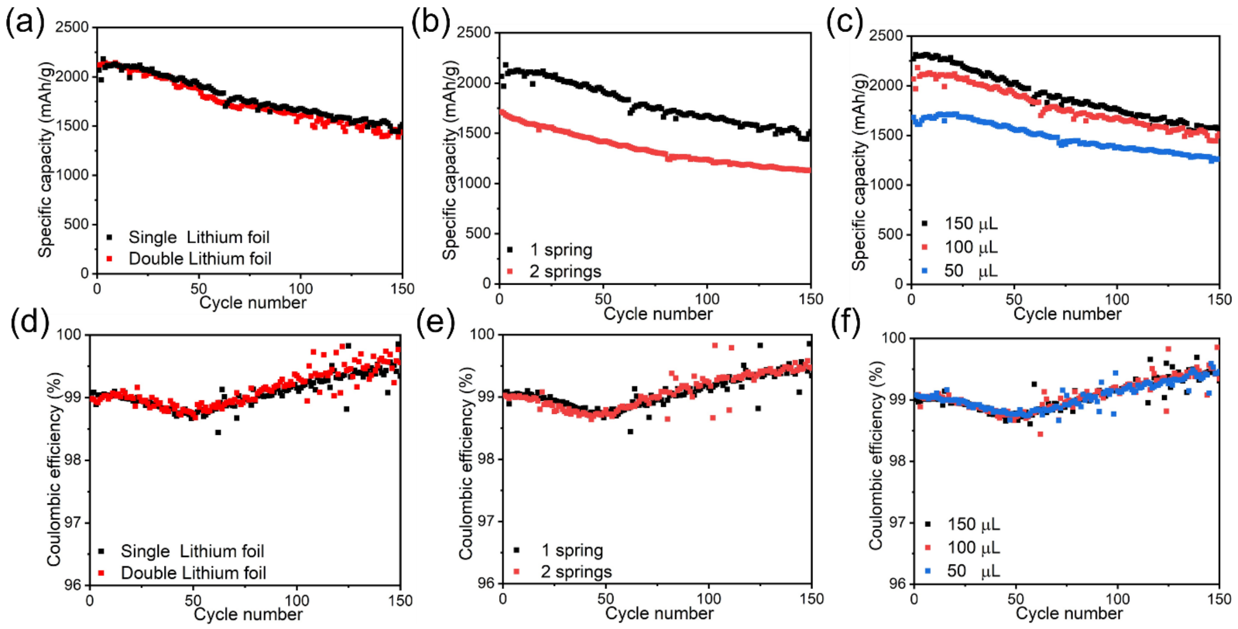

Figure S2. Specific capacity variations of Si anodes with (a) single and double lithium, (b) one and two springs, and (c) different electrolyte volumes. CE variations of Si anodes with (d) single and double lithium, (e) one and two springs, and (f) different electrolyte volumes.

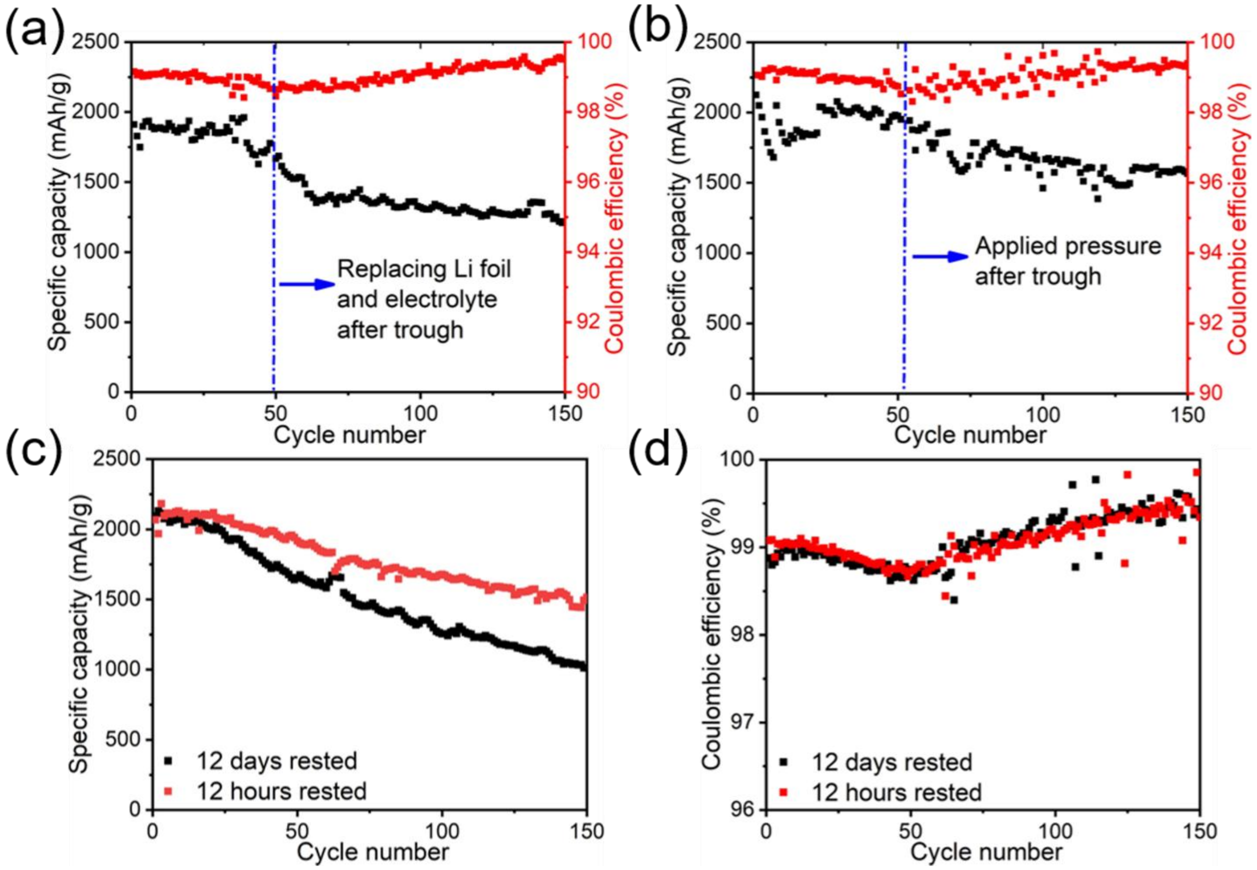

Figure S3. Specific capacity and CE of Si anodes (a) before and after replacing Li foil and electrolyte, (b) before and after applying external pressure. (c) Specific capacity of Si anodes after resting for 12 days and 12 hours. (d) CE of Si anodes after resting for 12 days and 12 hours.

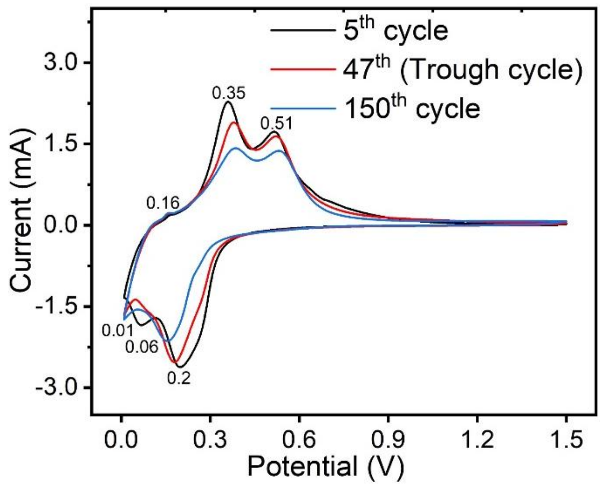

Figure S4. CV curves of Si anodes at the 5th, 47th (the CE trough cycle), and 150th cycles.

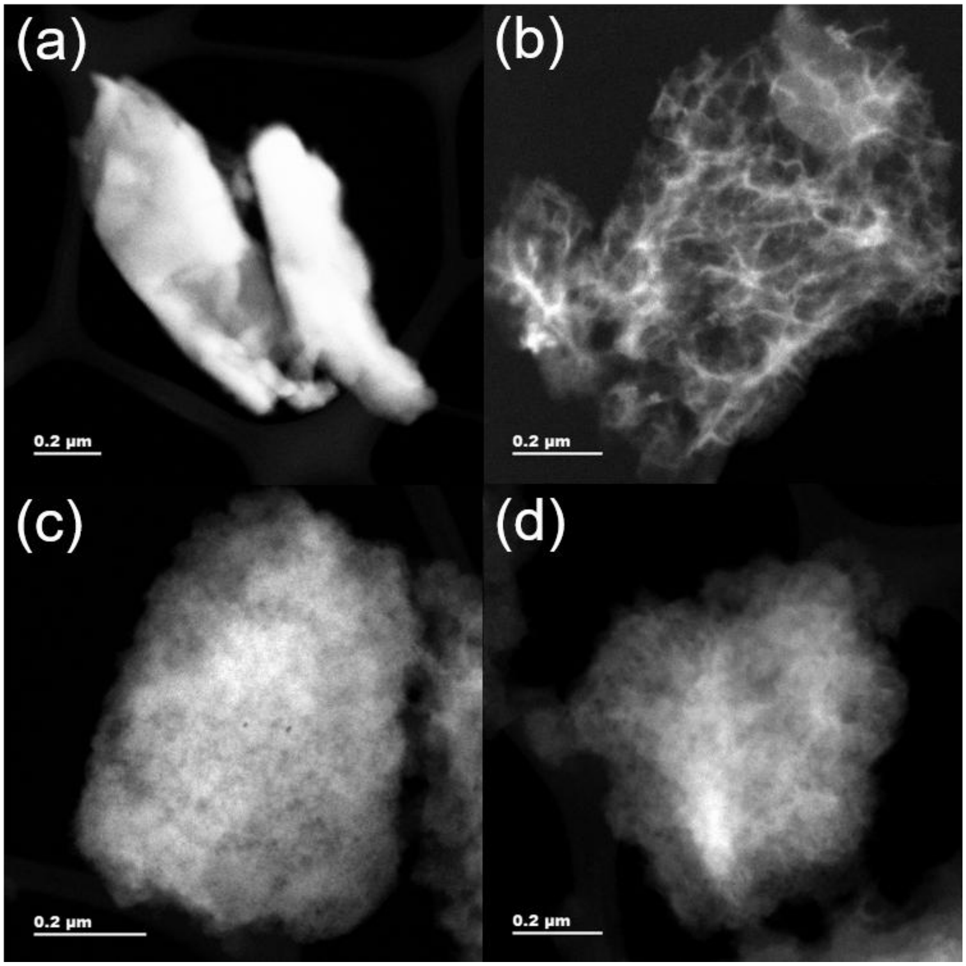

Figure S5. HAADF-STEM images of Si anodes (a) before cycling, (b) after the CE trough cycle with BE + FEC electrolyte and 0.01-1.5 V, (c) after the CE trough cycle with THF electrolyte and 0.01-1.5 V, and (d) after the CE trough cycle with BE + FEC electrolyte and 0.01-0.5 V.

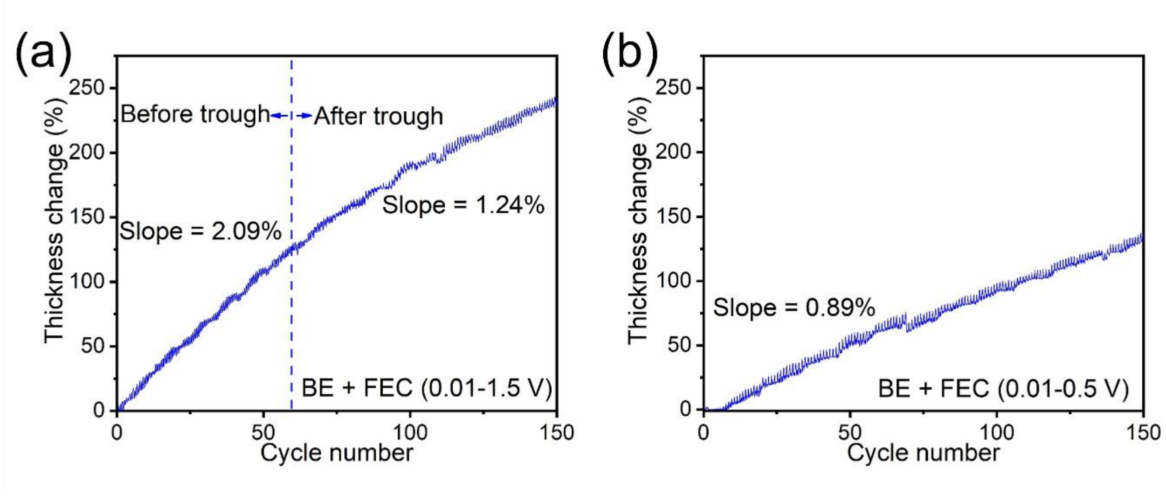

Figure S6. Dilatometer data of Si anodes cycled under (a) 0.01-1.5 V and (b) 0.01-0.5 V voltage windows with BE + FEC electrolyte.

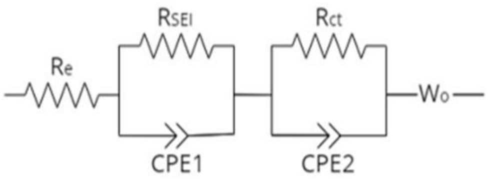

Figure S7. The equivalent circuit model for EIS fitting.

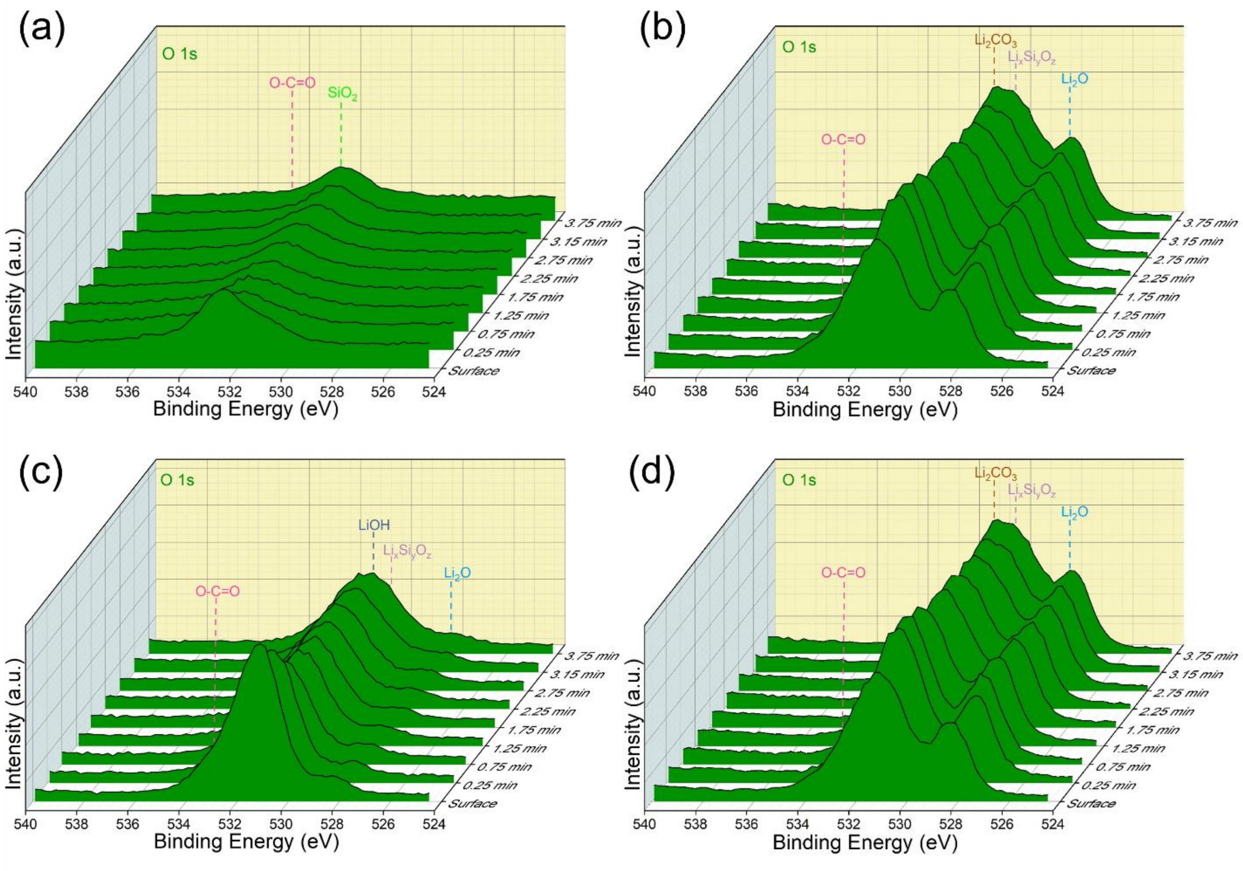

Figure S8. XPS O 1s depth profiles of Si anodes (a) before cycling, at the CE trough cycle with (b) BE + FEC electrolyte and 0.01-1.5 V, (c) THF electrolyte and 0.01-1.5 V, and (d) BE + FEC electrolyte and 0.01-0.5 V.

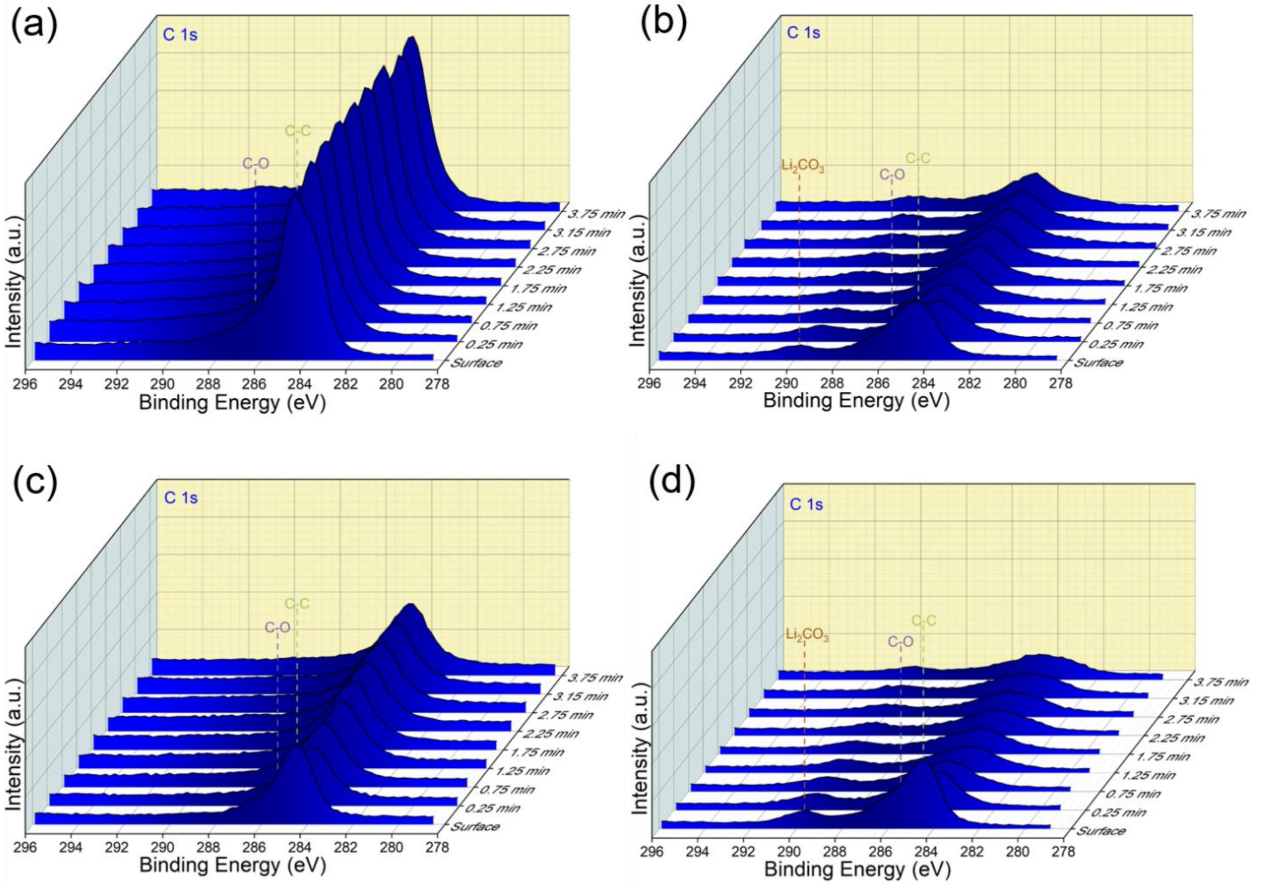

Figure S9. XPS C 1s depth profiles of Si anodes (a) before cycling, at the CE trough cycle with (b) BE + FEC electrolyte and 0.01-1.5 V, (c) THF electrolyte and 0.01-1.5 V, and (d) BE + FEC electrolyte and 0.01-0.5 V.

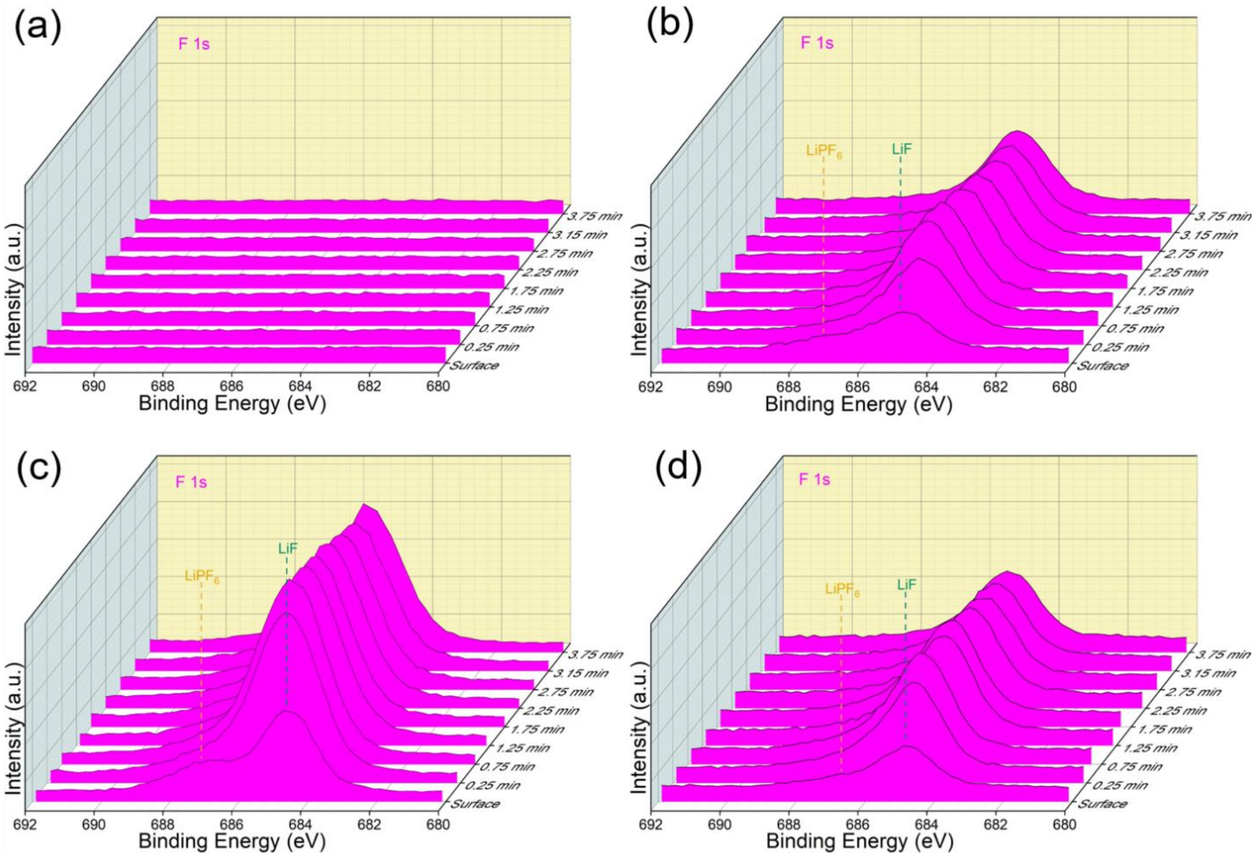

Figure S10. XPS F 1s depth profiles of Si anodes (a) before cycling, at the CE trough cycle with (b) BE + FEC electrolyte and 0.01-1.5 V, (c) THF electrolyte and 0.01-1.5 V, and (d) BE + FEC electrolyte and 0.01-0.5 V.

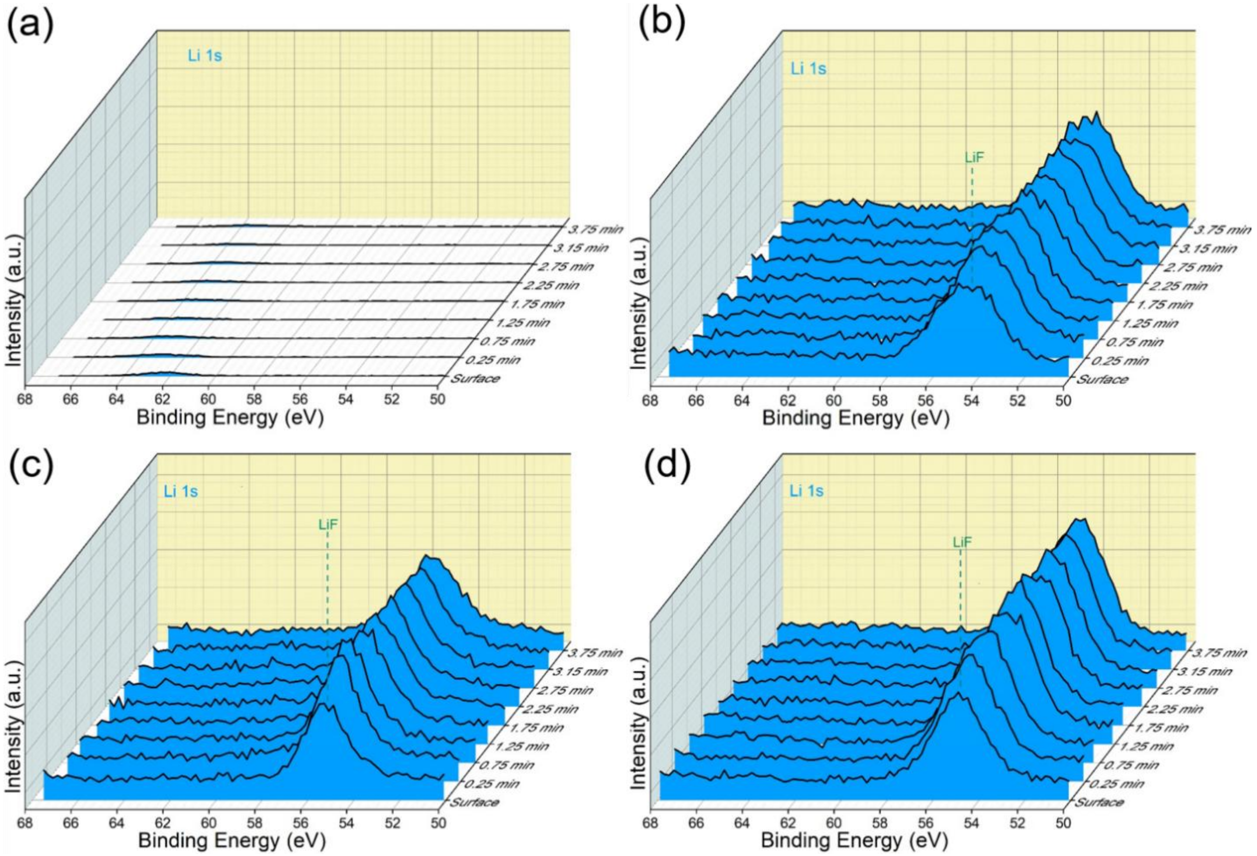

Figure S11. XPS Li 1s depth profiles of Si anodes (a) before cycling, at the CE trough cycle with (b) BE + FEC electrolyte and 0.01-1.5 V, (c) THF electrolyte and 0.01-1.5 V, and (d) BE + FEC electrolyte and 0.01-0.5 V.

Table S1. Comparison of silicon-based anode studies, highlighting key parameters, findings, and CE trough observation.

Anode Material	Electrolyte Formulation	Voltage window (V)	CE trough cycle # & value (%)	Key Findings	Ref
Silicon-graphite	1.0 M $LiPF_6$ in EC and EMC (3:7 w/w) plus 5 wt% FEC	0.01-1.25	18th (~97.4)	Examining silicon-graphite electrodes (20–60 wt% Si), the focus was on capacity degradation without addressing efficiency losses.	[1]
Silicon-graphite	1.0 M $LiPF_6$ 1:1 (v/v) EC and DMC with 0.1, 0.5, or 2.0 vol% FEC	0.0-0.5<br>0.0-0.7<br>0.0-1.0<br>0.0-1.5<br>0.0-2.0	No Trough<br>135th (~97.7)<br>79th (~96.4)<br>99th (~96.4)<br>115th(~96.2)	Investigating FEC consumption, the analysis centered on its role in EC and DMC decomposition and SEI formation, emphasizing the working potential range.	[2]
Porous Si	1.0 M $LiPF_6$ in (EC:DEC = 1:1, v/v) with a 10% FEC	0.005-1.5	32th (~98.5)	Exploring mesoporous silicon particle size, the study assessed its impact on morphology and electrochemical performance.	[3]
Silicon-graphite	1.0 M $LiPF_6$ in (EC) and (EMC) (3:7 w/w) plus 5 wt% FEC	0.01-0.65<br>0.01-1.25<br>0.05-1.25	No Trough<br>40th (~97.5)<br>50th (~97.6)	Showing that silicon-graphite degradation stems from porosity, SEI growth, and swelling, stabilization was observed after ~60 cycles, mitigated by limiting delithiation to 0.65 V.	[4]
Si nanowire	1.2 M $LiPF_6$ in (EC:DMC = 3:7, v/v) with a 10% FEC	0.02-1.5	45th (~98.4)	Identifying void formation and electrolyte permeation as key drivers of capacity decay, the analysis provided insights into material instability.	[5]
Porous Si	1.0 M $LiPF_6$ in (EC:DEC = 1:1, v/v) with a 5% FEC	0.01-1.5	70th (~98.4)	Demonstrating a scalable method for nanoporous Si synthesis, the focus remained on fabrication without long-term electrochemical evaluation.	[6]
Pomegranate-nano-Si	1.0 M $LiPF_6$ in (EC: DEC = 1:1, v/v) with a 1% VC additive	0.05-1	45th (~98.6)	Introducing a biomimetic pomegranate-inspired structure, the design aimed to address silicon expansion and SEI formation.	[7]
Silicon/carbon composite	1.0 M $LiPF_6$ in (EC:DMC 1:1, v/v) with a 10 % FEC	0.01-1.5	19th (~92.5)	Focusing on cycling stability, the evaluation considered buffer solutions, binders, and constrained cycling strategies.	[8]
Micron-Si/graphene	1.0 M $LiPF_6$ in EC/EMC = 1:2 in vol + 25 vol % FEC	0.01-1.5<br>0.01-0.5<br>0.1-1.5	47th (98.6)<br>No Trough<br>118th (98.8)	Directly examining CE trough, its mechanisms, and mitigation strategies.	**This work**
	2.0 M $LiPF_6$ in THF/2mTHF = 1:1 in vol	0.01-1.5	No Trough		

Table S2. Summary of factors tested for their impact on the CE trough in Si anodes.

Factor	Affecting CE Trough	Comment
Areal density (mg cm^{-2})	Yes	Lower areal density leads to an earlier CE trough
Packing density (g cm^{-3})	No	No impact
Si loading (%)	Yes	Less active material results in a smaller CE trough
Particle size (μm – nm)	Yes	The lowest CE in nano-Si occurs at the beginning
C-rates	Yes	Lower C-rates lead to an earlier CE trough
Li foil quantity	No	No impact
Spring quantity	No	No impact
Electrolyte volume	No	No impact
Li foil freshness	No	No impact
Electrolyte freshness	No	No impact
External pressure	No	No impact
Rest	No	No impact

References

[1] M. Wetjen, D. Pritzl, R. Jung, S. Solchenbach, R. Ghadimi, H. A. Gasteiger, *J. Electrochem. Soc.* **2017**, *164*, A2840.
[2] S. Yamazaki, R. Tatara, H. Mizuta, K. Kawano, S. Yasuno, S. Komaba, *J. Phys. Chem. C* **2023**, *127*, 14030.
[3] W. Cao, K. Han, M. Chen, H. Ye, S. Sang, *Electrochim. Acta* **2019**, *320*, 134613.
[4] M. Wetjen, S. Solchenbach, D. Pritzl, J. Hou, V. Tileli, H. A. Gasteiger, *J. Electrochem. Soc.* **2018**, *165*, A1503.
[5] Y. He, L. Jiang, T. Chen, Y. Xu, H. Jia, R. Yi, C. Wang, *Nat. Nanotechnol.* **2021**, *16*, 1113.
[6] W. C. Cho, H. J. Kim, H. I. Lee, M. W. Seo, H. W. Ra, S. J. Yoon, J. W. Choi, *Nano Lett.* **2016**, *16*, 7261.
[7] N. Liu, Z. Lu, J. Zhao, M. T. McDowell, H. W. Lee, W. Zhao, Y. Cui, *Nat. Nanotechnol.* **2014**, *9*, 187.
[8] H. F. Andersen, C. E. L. Foss, J. Voje, R. Tronstad, T. Mokkelbost, P. E. Vullum, J. P. Mæhlen, *Sci. Rep.* **2019**, *9*, 1.
